# Dynamical stochastic simulation of complex electrical behavior in neuromorphic networks of metallic nanojunctions

**DOI:** 10.1038/s41598-022-15996-9

**Published:** 2022-07-18

**Authors:** F. Mambretti, M. Mirigliano, E. Tentori, N. Pedrani, G. Martini, P. Milani, D. E. Galli

**Affiliations:** 1 CIMAINA and Dipartimento di Fisica, Università degli Studi di Milano, via Celoria 16, 20133 Milano, Italy; 2 Dipartimento di Fisica e Astronomia, and INFN - Sezione di Padova, Università degli Studi di Padova, via Marzolo 8, 35131 Padova, Italy

**Keywords:** Nanoscale materials, Nanoparticles, Materials science, Nanoscale materials, Electronic properties and materials

## Abstract

Nanostructured Au films fabricated by the assembling of nanoparticles produced in the gas phase have shown properties suitable for neuromorphic computing applications: they are characterized by a non-linear and non-local electrical behavior, featuring switches of the electric resistance whose activation is typically triggered by an applied voltage over a certain threshold. These systems can be considered as complex networks of metallic nanojunctions where thermal effects at the nanoscale cause the continuous rearrangement of regions with low and high electrical resistance. In order to gain a deeper understanding of the electrical properties of this nano granular system, we developed a model based on a large three dimensional regular resistor network with non-linear conduction mechanisms and stochastic updates of conductances. Remarkably, by increasing enough the number of nodes in the network, the features experimentally observed in the electrical conduction properties of nanostructured gold films are qualitatively reproduced in the dynamical behavior of the system. In the activated non-linear conduction regime, our model reproduces also the growing trend, as a function of the subsystem size, of quantities like Mutual and Integrated Information, which have been extracted from the experimental resistance series data via an information theoretic analysis. This indicates that nanostructured Au films (and our model) possess a certain degree of activated interconnection among different areas which, in principle, could be exploited for neuromorphic computing applications.

## Introduction

The expression ‘neuromorphic computing’ is a roof under which are gathered several hardware and software approaches aiming at overcoming the difficulties of digital computers to respond to the continuously increasing demand for complex data processing at low energetic cost^1–8^. This ambitious objective can be summarized in the reproduction of the mammalian brain capabilities of rapidly integrating information from many different sources^9^. Mammalian brains are composed by an extremely high number of electrically active neurons; the synaptic weights regulate the interconnection between neuron pairs and also define the network topology, as they control the propagation of signals across the synapses^10^. These weights are not fixed but can change over time depending on their previous history, thus providing the learning capability of the network. The organization of structural connections among different regions of the brain largely determines the types of cognitive functions that can be supported, including memory, learning, vision and motor control^10^. Artificial neuromorphic systems should be composed by building blocks able to emulate the properties of their biological counterparts: neurons, synapses, axons and dendrites, wired with an extremely high degree of inter-connectivity.

The memristor is a non-linear device whose electrical properties are dependent on the history of the current and/or voltage it has experienced^11^; this results in two important synapse-like properties: plasticity and retention^11,12^. Memristors have been successfully embedded into various CMOS architectures, typically organizing them as large arrays or 3D stackings to fabricate artificial systems with neuromorphic behavior^12,13^. By controlling the internal state of single memristors (nodes), memorization, learning and classification can be obtained^13,14^.

An approach to synthetic neuromorphic networks gaining increasing attention, is represented by the self-assembling of nanoscale building blocks like nanowires^15–17^, and nanoparticles^18^ to form complex networks of interconnected nanoscale electrical switching elements that exhibit synapse-like behavior^16,17,19,20^ and mimic the complex network topology of neurons in the brain^21,22^.

In contrast to thin-film memristors^20^, the conductance state of a random assembly of resistive switching junctions does not only depend on the dynamics of a single element, but also on the topological organization of the connected nanoobjects^23^. The interplay between junction dynamics and network topology produces complex effects, such as self-organization and criticality, also encountered in neuronal systems^24,25^ with potential applications in reservoir computing^26^. This subtle balance between the non-linear, memristive-like electrical properties of nano-objects networks and their topological organization has been recognized as a fundamental aspect of the observed neuromorphic behavior^19,23,27^, in particular, the connection between the topological organization and information processing is critical for performing memory and learning tasks^22,23,28^. Despite these fundamental progresses, the influence of the nanoscale switching mechanism on the collective electrical properties at different length scales remains elusive^19,23^. Due to the complexity of networks obtained by the self-assembling of nano-objects, it is very difficult to predict the impact of the network structure on their data processing capabilities. Among the computational approaches which tackle this issue (see e.g.^19,29,30^), we recall here that state-of-the-art simulations and analysis of information dynamics in micrometric nanowire electrical networks are reported in^31,32^.

**Figure 1:**
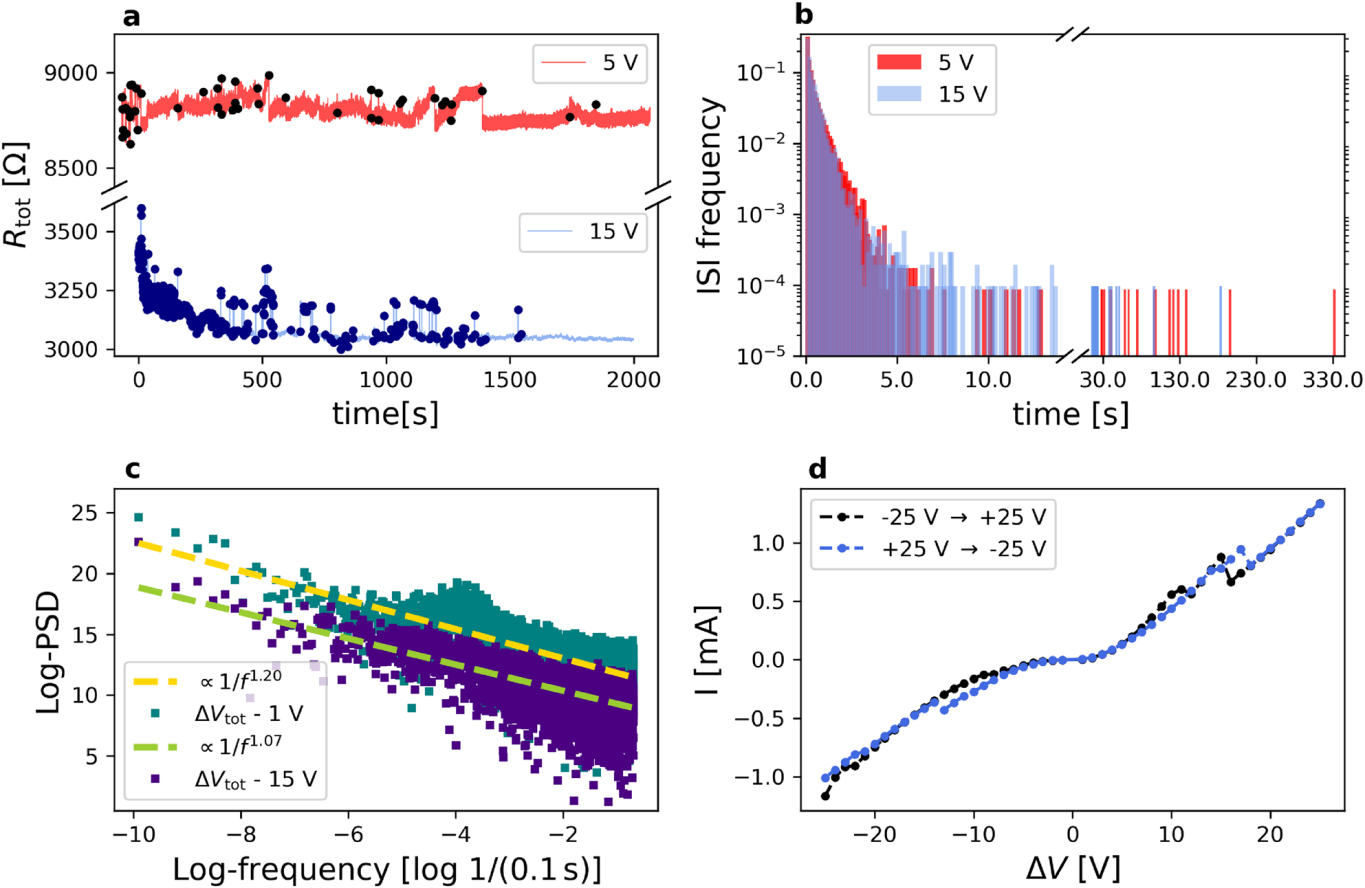
(**a**) typical evolution of electrical resistance with time under the application of ${\Delta V} =5\ \hbox {V}$(red) or 15 V (lavender), measurements are taken every 100 ms. Black and dark blue dots correspond to resistance switches. (**b**) Inter-Switch-Interval probability density, plotted collecting the temporal distance $t_{\mathrm{ie}}=t_{i+1}-t_i$ (the inter-event time) of consecutive switches. (**c**) Experimental PSD for measurements taken at 1 V (blue-blue) and at 15 V (dark purple), as a function of frequency *f*, in log-log scale. Yellow and blue dashed lines represent $1/f^{\alpha }$ fitted curves. (**d**) apparently nonlinear $I(\Delta V)$ curve, from -25 V to +25 V, with discrete jumps equal to 1 V.

Recently we showed that gold nanostructured films resulting from the assembling of metallic clusters show complex resistive switching activity, non-linear dynamics, capacity of learning, with the emergence of spatially correlated structures of network activity^24,33–35^. The switching activity and its dependence from the flow of electrical current through the nanostructured metallic film^36^ can be used for neuromorphic computing applications^24,35,37^. The nanogranular structure of cluster-assembled Au films consists of an extremely high density of nanojunctions with memristive-like behavior^24,37^. Correlations emerge among the electrical activity of different regions of the film under the application of an external electrical stimulus higher than a suitable threshold. The degree of correlation can be varied controlling the film geometry and the electrode configuration used as input and output^37^. In Fig. 1a we display two typical measured total resistance series in time, $R_{\mathrm{tot}}(t)$, characterized by the onset of switching activity with a series of discrete and reversible resistance variations, following the application of an external voltage bias $\Delta V$. As presented and discussed in detail in^24^ the data refers to cluster-assembled films characterized by different initial resistances: in the case of high initial resistance, we observe the onset of a resistive switching activity upon the application of low constant voltages (3 V). At 5 V the high resistance films show a resistive switching pattern with a small number of events uniformly distributed (red dots). In the case of low resistance films, we observe the presence of a pronounced switching activity from roughly 15 V (blue dots)^24^. No decreasing of switching activity has been observed as reported in^24,33,57^. In Fig. 1a resistance data show an initial decreasing trend. This is due to joule heating effect arising from the current flowing through the metallic film used to trigger switching activity^33^. The concurrent effects of local rearrangement phenomena, electromigration and joule heating acting on the cluster assembled of films and resulting in the structural reorganization of the cluster-assembled films have been discussed in^24,33,35,37^. Fig. 1b contains the Inter-Switch-Interval (ISI) distributions of the total resistance at two different voltages (5 V and 15 V), which have been obtained by collecting data from measurements on few samples produced and analyzed under analogous experimental conditions. The majority of the RS events are within an interval of 2 s and the ISI distributions are characterized by an approximately exponential decay with very extended tails of outliers. Previous investigations of ISI distributions in nanoparticle networks have shown different types of trends, including exponential, power-law and lognormal functions^54–56^. Figure 1c reports, instead, the measured Power Spectral Density of $R_{\mathrm{tot}}(t)$ for two other series at different voltages (1 V and 15 V). The measured electrical signal is characterized by temporal correlations with a Power Spectral Density (PSD) featuring a $1/f^{\alpha }$ trend, being $1< \alpha < 1.5$. This behavior is typical of systems where memory effects are present^24,25^. The non-linear electrical behavior of cluster-assembled Au films is also highlighted by the $I(\Delta V)$ curves reported in Fig. 1d^24,33,35^.

Cluster–assembled gold films are characterized by the presence of a high density of structural defects such as grain boundaries and dislocations: this causes the departure from an ohmic behavior and the appearance of non–local effects^24,33,34,37^. Structural defects are responsible for variation of the local resistance and hence of formation of nanoscale regions where the local temperature varies in a very wide range of values, depending on the value of the resistance^38^. This causes defect migration, grain boundaries modification and annealing, local re–crystallization phenomena depending to the different nanoparticle dimensions and structures^24,34,37^. The nanostructured film dynamically reorganize in different local crystalline structures following the evolution of the temperature distribution caused by the change in local conductivity^19^. These effects cause a dynamical redistribution of the flowing current through the network of nanojunctions and the resistive switching activity^24,34,37^.

With the aim to capture the necessary minimal complexity which can effectively describe, at a coarse-grained level, the electrical transport phenomena characterizing nanostructured Au films, we have thus conceived a Stochastic Resistor Network (SRN) model. This model couples a stochastic dynamics mimicking various physical effects, like thermal dissipation and topological reorganization of the nanogranular structure (and the related electrical transport properties, quantum effects included)^24,35^, to a three-dimensional regular lattice of resistors with discrete conductances. Other stochastic approaches have previously appeared in literature. Simple two-dimensional models based on resistor networks, like the Random Circuit Breaker (RCB) network model, have been implemented for a microscopic description of (unipolar) resistive switching phenomena^40,41^ in the context of Resistive Random Access Memory devices, whereas stochastic dynamic versions of the RCB model have been also developed^42,43^. Another stochastic framework present in literature is a continuum percolation model^53,54^, with static probabilities of formation and breaking of atomic scale wires in 2D, which is able to generate avalanches of switching events similar to potentiation mechanism in biological neural systems. More recently, the deterministic evolution of random nanowire networks employed for neuromorphic computing applications has been simulated at a micrometric scale^31,32^.

The peculiarity of our SRN model is that some of the stochastic moves, which involve single resistor updates, are influenced by the dissipation process of the neighboring resistors, inducing mutual dependencies among the simple network elements. The crucial novelty of this approach lies therefore in the attempt to retrieve the key features of our complex experimental phenomenology starting from a large 3D resistor network that stochastically evolves due to the cooperativity of its building blocks, despite each of them can access only a limited number of resistance states. Remarkably, beyond a given lattice size, the model gains enough complexity to be able to display resistive switching, a nontrivial PSD frequency dependence and a nonlinear $I(\Delta V)$ behavior. Moreover, exploiting entropy-based tools typical of neuroscience for measuring brain complexity, the experiment and our SRN model both highlight peculiar non-local spatial correlations, displaying many common features with biological neuromorphic systems^21,39^.

## Results

### A coarse-grained Stochastic Resistor Network model

**Figure 2:**
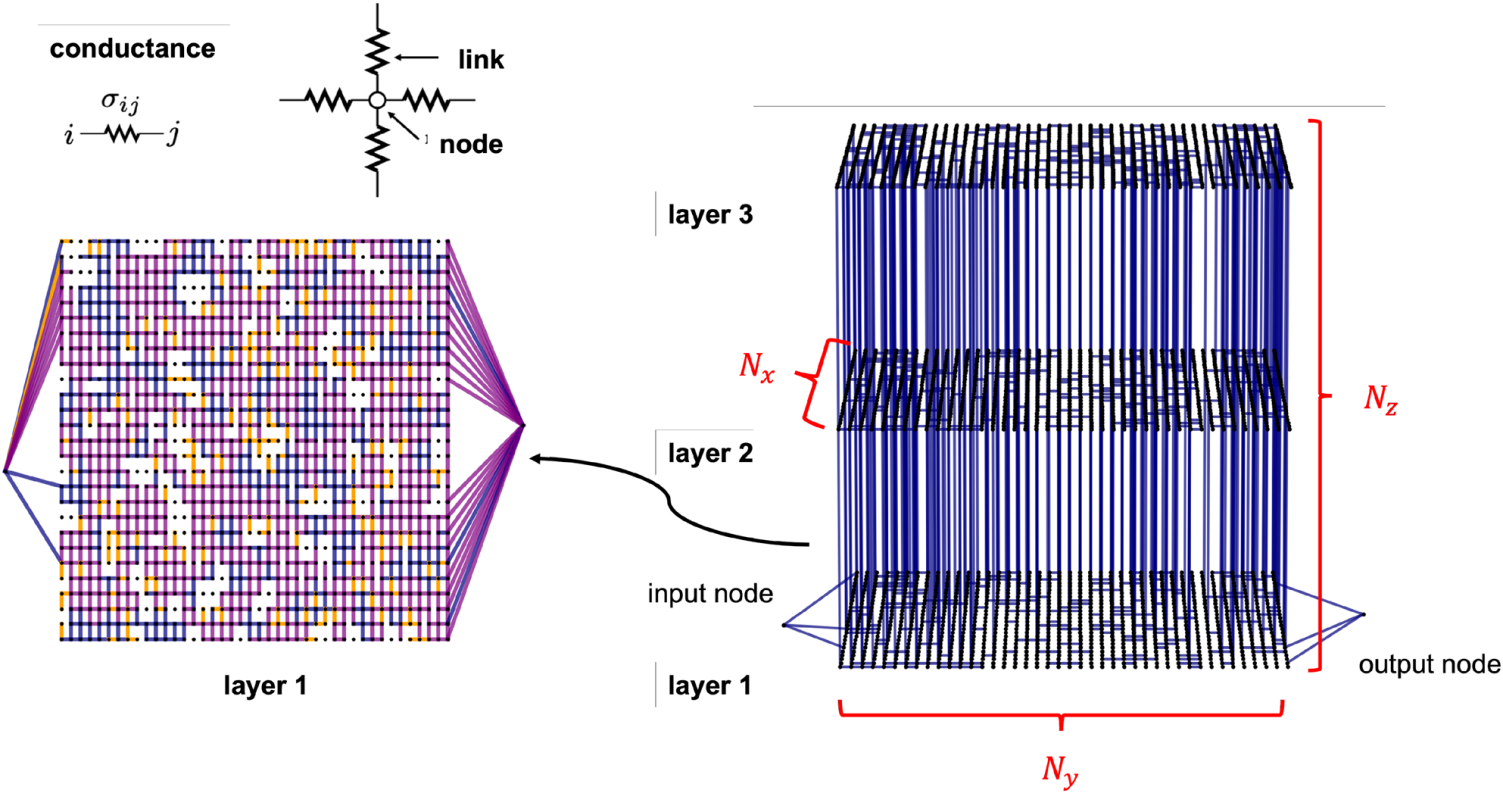
Left: schematization of a node, a link and viem of the layer 1 of the resistor network. Each link can assume one of a discrete number of conductance values (namely, in this work, $\sigma _{\alpha }=10^{-11} \frac{1}{\Omega }$, $\sigma _{\beta }=10^{-3} \frac{1}{\Omega }$, $\sigma _{\gamma }=2 \times 10^{-3} \frac{1}{\Omega }$ and $\sigma _{\delta }=4 \times 10^{-3} \frac{1}{\Omega }$). In the picture, links with $\sigma _{ij}=\sigma _{\alpha }, \sigma _{\beta }, \sigma _{\gamma }, \sigma _{\delta }$ are respectively white, orange, blue and purple. Right: 3D view of the full network, where only edges with $\sigma _{ij}=\sigma _{\gamma }$ are colored. $N_x, N_y, N_z$ correspond to the number of nodes along each network direction. Picture realized with NetworkX^44^.

Aiming to describe the electrical behavior of macroscopic cluster-assembled metallic films at a coarse-grained level, our Stochastic Resistor Network model is essentially based on a large three-dimensional (3D) regular lattice of resistors, each one capable of a discrete number of conductive states, which in our work has been fixed to four (see Fig. 2 and Methods). This choice is a trade-off between descriptive capability and the need to limit the complexity of our simulation framework. Resistors are represented as *links* *ij* joining a pair of *nodes* (*i*, *j*), which are coarse-grained representations of large sub-regions of the original sample; the links are organized in groups termed *layers* (which are three in Fig. 2), connected to each other by vertical (*z*-axis) links. The conductance values were established based upon experimental data on nanostructured Au films. As shown in Fig. 2 our resistor network is provided with an input and an output node, to which an overall voltage $\Delta V$ is applied.

For each link connecting nodes *i* and *j*, the applied voltage induces the presence of electrical current $I_{ij}$ flowing through it, and also a potential difference $\Delta V_{ij}$. Applying the spectral theory to the Laplacian matrix of the weighted undirected graph associated to our network^31,32,45–48^, we are able to retrieve $I_{ij}$ and $\Delta V_{ij}$ for each link of the network. The set of these $I_{ij}$ and $\Delta V_{ij}$ values constitutes the input for Monte Carlo update moves which make the system stochastically evolve. These MC moves are conceived to reproduce the thermal stability of the connections and also other non-linear electron conduction mechanisms, due to inter-cluster and intra-cluster atomic rearrangements, which result in the dynamical creation and destruction of conduction pathways and trigger the switching events^24,33–35^. Such physical effects are reflected in our model through the possibility for each link *ij* to either increase its own conductance or decrease it, via stochastic jumps across the available discrete conductance levels, due to the heat released by its neighbors ($\sigma _{ij}$ grows with a given probability) or to its thermal dissipation ($\sigma _{ij}$ lowers with a given probability), respectively—see Methods. Besides, $\Delta V_{ij}$ is nonlinearly used either to stochastically degrade or improve $\sigma _{ij}$, by comparing it with a suitable threshold $\Delta V^{\mathrm{th}}$ (see Methods for details). The computational cost of the model is highly demanding, since the application of the aforementioned stochastic update moves produces a new configuration of the network, which then requires a new complete solution in terms of currents $I_{ij}$ and voltages $\Delta V_{ij}$ at each link, to provide the input for the subsequent MC step. In view of the computational effort required, our aim has been to find out and focus on a well-founded set of parameters capable of reproducing the experimental phenomenology.

### Qualitatively reproducing experimental electrical transport properties via the SRN model

**Figure 3:**
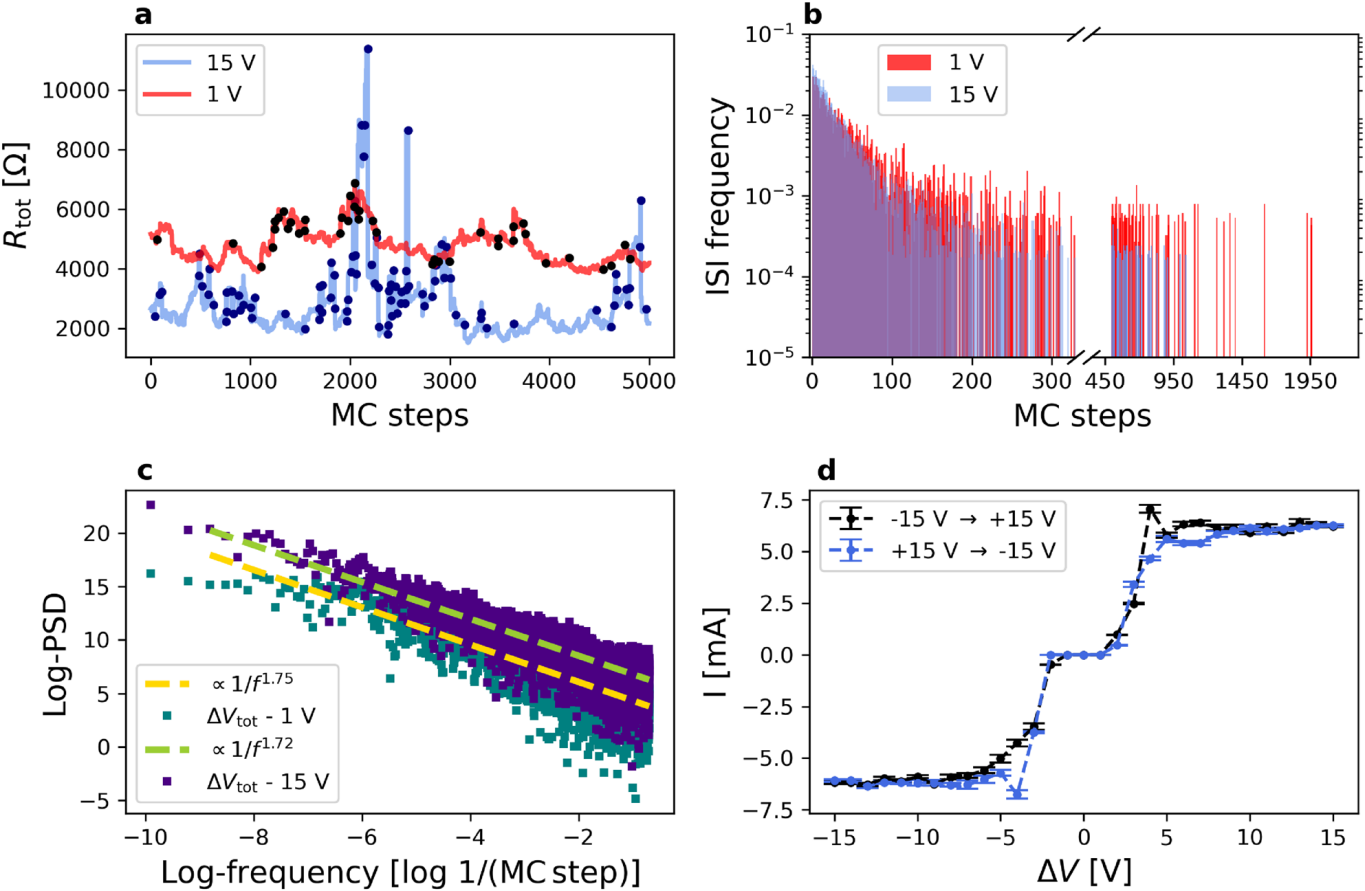
(**a**) typical evolution of simulated electrical resistance under the application of ${\Delta V} =1\ \hbox {V}$ (red) or 15 V (lavender); measurements are taken every MC step. Black and dark blue dots correspond to resistance switches. (**b**) Inter-Switch-Interval probability density, computed as for the experimental data, joining data from 10 statistically independent simulations. Also here, at 15 V the distribution is approximately monotonically decreasing and the majority of the RS events are close in time (within $\approx 1000$ MC steps). The data taken at 1 V feature a longer tail of the distribution, as in the experiments at low voltage. (**c**) Simulated PSD for measurements taken at 1 V (blue-blue) and at 15 V (dark purple), as a function of frequency *f*, in log-log scale. Yellow and blue dashed lines represent $1/f^{\alpha }$ fitted curves. **d**: simulated $I(\Delta V)$ curve, where each point is averaged over 20 independent simulations lasting 8000 MC steps each (the dashed line is only a guide for the eyes).

Our SRN model is capable of generating a rich and complex electrical transport phenomenology which is impossible to forecast in advance; nonetheless, the key ingredients were conceived in the attempt to qualitatively retrieve the experimental features of Fig. 1. Remarkably, we see the progressive emergence of all the peculiarities observed in the electrical conduction properties of nanostructured gold films, as long as the network size is gradually increased. In particular, the size of our regular 3D lattice has been progressively enlarged, up to the size of $N_x=27 \times N_y=42 \times N_z=3$, which corresponds to 3404 nodes and 8919 links. The simulation of such a large network, endowed with a nontrivial stochastic dynamics, represents an unprecedented attempt to study the complexity required to describe electrical conduction phenomena in cluster–assembled nanostructured metallic systems. Our results thus indicate the first identification of a minimum complexity limit to be considered in order to start achieving such an experimental phenomenology. The simulations of our SRN model have required an extensive use of high performance computating facilities. Despite this size and the resulting intricacy, our SRN model cannot capture the full complexity of the experimental system; for this reason we can only expect a qualitative reproduction of the phenomenology observed in gold nanostructured films. We also point out that, keeping the potential difference $\Delta V$ fixed, the effect of rescaling by a constant factor, $\Gamma$, all the conductances, $\sigma _{ij}$, and correspondingly, adequately rescaling all the model activation thresholds, has as its only effect an equivalent stochastic dynamics of the model, characterised by the same $\Delta V_{ij}$ with currents $I^{\prime }_{ij} = \Gamma \times I_{ij}$. We show in Fig. 3 the analogue quantities displayed in Fig.1, simulated via the SRN model.

Figure 3a shows the evolution of ${R_{\mathrm{tot}}}$ during a typical simulation of 20000 MC steps (where a MC step is the simulation basic time unit) at $\Delta V = 15 V$ for our SRN model. The analysis of the resistance series data for two-electrode devices (see Fig. 7, Methods section) is carried out following the same protocol used for the experimental data. The qualitative similarity between the experimental and simulated patterns suggest that the SRN model is able to capture the main features evidenced in the experiment: a number of distinguished resistance levels are repeatedly visited by the system with sudden variations of ${R_{\mathrm{tot}}}$ and subsequent fluctuations around a given resistance value. In the stochastic evolution of ${R_{\mathrm{tot}}}$ we observe more intense fluctuations with respect to the experimental case; the reason for this lies, as discussed below, in the lower complexity of the SRN model compared to the real system and the consequent greater fragility and susceptibility of ${R_{\mathrm{tot}}}$ to the stochastic dynamics.

We evaluated the Inter-Switch-Interval distribution of ${R_{\mathrm{tot}}}$ and the the Power Spectral Density of $R_{\mathrm{tot}}$, i.e. the square modulus of the Fourier transform of the signal. The Power Spectral Density of the measured electrical signal for our SRN model is characterized by a $1/f^{\alpha }$ trend^24^, with typical $\alpha$ values between 1.5 and 2. ISI distribution is obtained like in the experimental data analysis. The data are plotted in form of probability density distribution function in Fig. 3b for devices polarized at 15 V. Notably, the ISI distributions coming from the SRN model are characterized by the same trend obtained from the experimental data. Even for the SRN model, at short times the data decrease like an exponential with similar timescales for both voltages, while extended tails of outliers are present, as also found in the experiments (see Fig. 1b). The majority of the RS events are most frequently separated by less than 70 MC steps, with very long tails of the distributions for larger MC steps intervals. The comparison of the experimental and simulated ISI distributions allows us to roughly associate to a single MC step an equivalent time, for the observed phenomenology, of the order of 0.1 seconds.

In Fig. 3c, a typical PSD of the SRN total resistance is reported in log-log scale, for simulations performed at 1 V and at 15 V (lavender and dark read points, respectively). Dashed dark blue and pale red lines represent power-law fits of the simulated data, with exponent $\alpha$. Surprisingly, the simulated PSD associated to $R_{\mathrm{tot}}$ shows a $1/f^{\alpha }$ behavior with $1<\alpha <2$ (in the particular example shown, $\alpha =1.74$ and $\alpha =1.72$ at 1 V and and at 15 V respectively). This behavior evidences a pink-noise memory^25^, qualitatively similar to the noise generated by the cluster-assembled films studied in the experiments^24,35^.

Our SRN model presents a peculiar $I(\Delta V)$ relation, featuring very small current values at small voltages, a markedly different regime with a steep increase in *I* at intermediate voltages, and a saturation regime at higher voltages (see Fig. 3d). The regions at low and intermediate $\Delta V$ qualitatively resemble the experimental behavior displayed in Fig. 1d. Despite its considerable number of links and nodes, the SRN model unveils finite size effects at high voltages: the limited size of the network, jointly with its collective and cooperative dynamics, yields a saturation in $|I(\Delta V)|$ values. Given the remarkable qualitative reproduction of the experimental phenomenology, our simulations could provide insights about the evolution and modification of the structure of cluster-assembled Au films induced by current flow and responsible for the observed switching behavior. We concentrate on the effect of high and low voltage application on the modification of the electrical behavior of different junctions^24,35^.

**Figure 4:**
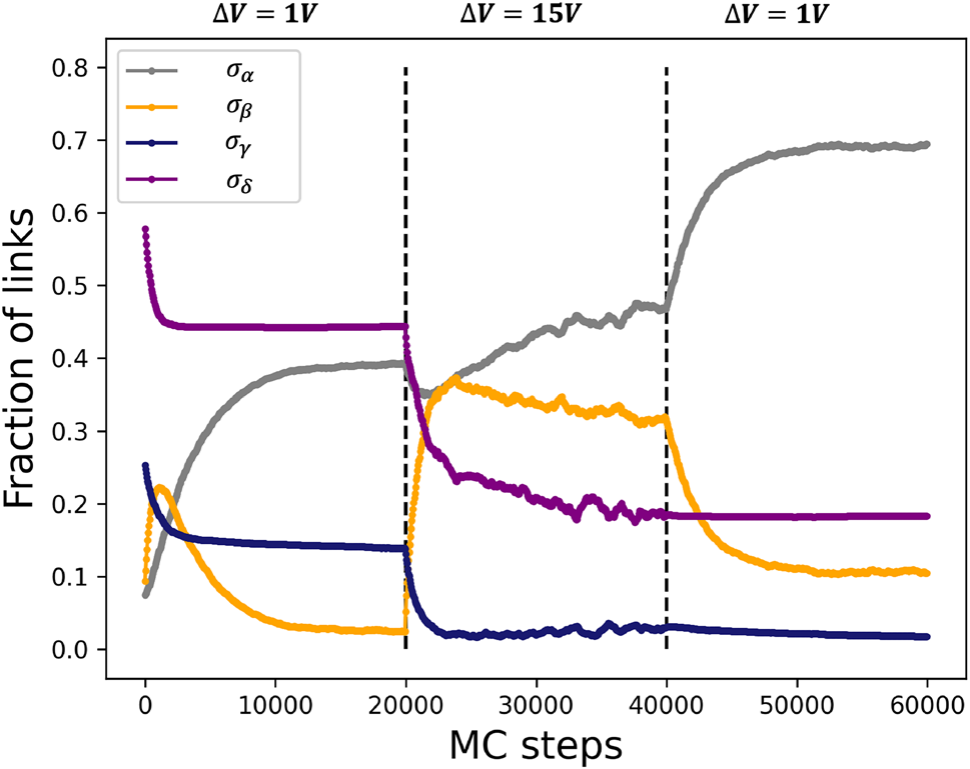
Evolution of the fractions of links with: $\sigma _{ij}=\sigma _{\alpha }=10^{-11} \Omega ^{-1}$ (grey), $\sigma _{ij}=\sigma _{\beta }=10^{-3} \Omega ^{-1}$ (orange), $\sigma _{ij}=\sigma _{\gamma }=2 \times 10^{-3} \Omega ^{-1}$ (navy) and $\sigma _{ij}=\sigma _{\delta }=4 \times 10^{-3} \Omega ^{-1}$ (purple) as a function of the MC steps. Black dashed vertical lines separate the three simulation phases. At the start of the simulation, one can see the initial probability for the abundance of links having conductivity equal to $\sigma _{\alpha }, \sigma _{\beta }, \sigma _{\gamma }, \sigma _{\delta }$. The initial amount of each conductance level is chosen in a range of values which allow for a sufficient degree of current percolation.

In Fig. 4, we report the evolution of the fractions of links having $\sigma _{ij}=\sigma _{\alpha }, \sigma _{\beta }, \sigma _{\gamma }, \sigma _{\delta }$ as a function of the MC steps, during a specific simulation where the effect of the application of a high voltage bias ($\Delta V=$15 V) to our resistor network has been studied. Initially, a low voltage is applied to a network featuring a purely random conductance distribution. First of all, we notice that, almost independently of the actual initial spatial distribution of the conductances, the system reaches a dynamical equilibrium as a consequence of the stochastic evolution of the model (see Fig. 4, slightly before 20000 MC steps). The application of a high voltage bias significantly alters this steady state, leading the system to a markedly different condition, characterized by a further decrease in the amount of the high conductances and a concurrent increase of the fraction of links having $\sigma _{ij}=\sigma _{\alpha }$.

In the subsequent phase, where $\Delta V= 1\hbox {V}$ again, the stochastic evolution leads it to the onset of a novel dynamical equilibrium (nonetheless characterized by the persistence of the resistive switching activity, see Fig. 3a), in close analogy with what is observed for the physical substrate: the number of highly resistive links almost doubles, corresponding to a picture where a significant reduction of the available paths for the current occurs. This is confirmed by the analysis of the shortest paths (measured weighting each link with the inverse of the current it is traversed by) available for the current from the input to the output, whose number is strongly reduced after that the system experiences a high voltage bias (see Supplementary Information). This result shows that the SRN model, whose stochastic dynamics is strongly correlated, is highly sensitive to the electrical conditioning history of the system, and supports the model based on local rearrangements of grain boundaries to explain the non-linear and non-local conduction properties of cluster-assembled Au films^24,35^.

### Information-theoretic analysis of correlations in the experimental device and the SRN model

In neuroscience, the role of dynamical correlations between different brain areas is recognized as fundamental for cognitive and behavioral integration^21,39^. Statistical measures derived from information theory have been proposed to characterize the integration of information among functionally segregated groups of neurons, in particular entropy, Mutual Information (MI) and Integrated Information (IN) have been considered to reveal the degree of interconnection/segregation for different regions of the brain or biological neural networks in response to external stimuli^39^.

Aiming to get a deeper insight into the presence and role of spatial correlations in cluster-assembled Au films, we performed an experimental analysis exploiting the same MI and IN tools^21,39^. To investigate the information content of the measured and simulated resistance series data, Mutual Information and Integrated Information have been computed as detailed below and in the Methods section. The nanostructured cluster films can indeed be idealized like networks of smaller units with a proper activity with a stochastic behavior. In literature different approaches are used to define the state of such a kind of system^22,49^. In a general approach, we can consider that the intensity of the electrical activity of each unit corresponds to different states of the elemental units.

Let us consider a generic set *X* of *N* Random Variables (RVs), whose subsets of size $k \le N$ are indicated as $X_j^k$, where *j* runs over all the $\dfrac{N!}{k!(N-k)!}$ possible choices of a subset of size *k*. Therefore, the elementary units of *X* can be referred to as $X_j^1, j=1 \dots N$. In the following equation, we give the definition of entropy $H(X_j^k)$ of a generic subset $X_j^k$, possibly even of $X=X_1^N$:

1 $$\begin{aligned} H (X_j^k) = \sum _i p_i (X_j^k) log_2(p_i (X_j^k)) \end{aligned}$$

being $p_i (X_j^k)$ the probability to find the subset $X_j^k$ in its *i*-th state. Exploiting the above general definition of entropy, two useful quantities can be introduced: the Mutual Information and the Integrated Information. MI takes into account the relationship between a subset $X_j^k$ and the complementary subset $X-X_j^k$, whereas IN includes the correlations among the basic units which are part of $X_j^k$^21,39^:

2 $$\begin{aligned}&\text {MI}(X_j^k,X-X_j^k)=\text {MI}(X-X_j^k,X_j^k)=H(X_j^k)+H(X-X_j^k)-H(X) \end{aligned}$$

3 $$\begin{aligned}&\text {IN}(X_j^k)=\sum _{l \, : X_l^1 \in X_j^k} H(X_l^1)-H(X_j^k) \end{aligned}$$

In Eq. (3), the index *l* runs over all the elements $X_l^1$ belonging to the subset $X_j^k$. MI describes the statistical dependence between the RVs represented by the subset $X_j^k$ of *X* and the complementary $X-X_j^k$. Note that MI$(X_j^k,X-X_j^k)=0$ if the two partitions of *X* are composed by independent RVs, while positive values of MI mark the presence of general statistical correlations between two data sets. Integration is, on the other hand, a tentative to give a measure of the segregation (independence) or integration (dependence) among the elementary constituents of the system. IN $(X_j^k)=0$ if the RVs composing the chosen subsystem, $X_j^k$, are statistically independent one from each other, while IN$(X_j^k)>0$ in presence of correlations among them.

Note that, in principle, one can compute also the average value of MI and IN:

4 $$\begin{aligned}&\overline{\text {MI}}_k :=\dfrac{k!(N-k)!}{N!}\, \sum _j \text {MI}(X_j^k,X-X_j^k) \end{aligned}$$

5 $$\begin{aligned}&\overline{\text {IN}}_k :=\dfrac{k!(N-k)!}{N!}\, \sum _j \text {IN}(X_j^k) \end{aligned}$$

In the case of our multi-electrode cluster-assembled films^37^, the system *X* can be thought of as made by elementary units $X_j^1$, which are single electrode couples (see Methods, and especially Fig. 7b for a system with $3 \times 3=9$ possible electrode pairs), where an electrode couple includes one of the three input terminals (1,2,3) together with one of the three output ones (A, B, C). The probability that the two electrodes selected have a given electrical resistance represents instead the basic ingredient from which we aim to infer entropy-related properties. Computing entropy for a set of *k* electrode couples, $H(X_j^k)$, using Eq. (1) requires having at disposal an approximated probability distribution $\{p_i\}$, where a probability is associated to a state *i*, corresponding to a list of *k* resistances simultaneously taken by all the involved electrode couples. More in detail, the time series of the resistance between each pair of electrodes is measured for a set of times ${\{t\}}$; each resistance time series $R_{\{t\}} (X_j^1)$ is normalized dividing by its mean value $\bar{R}(X_j^1)$ and then discretized (see Methods). The entropy related to a set $X_j^k$ of electrodes (and MI and IN as a consequence) can be then obtained computing the joint probability of the resistance states of the *k* electrode couples belonging to the set.

**Figure 5:**
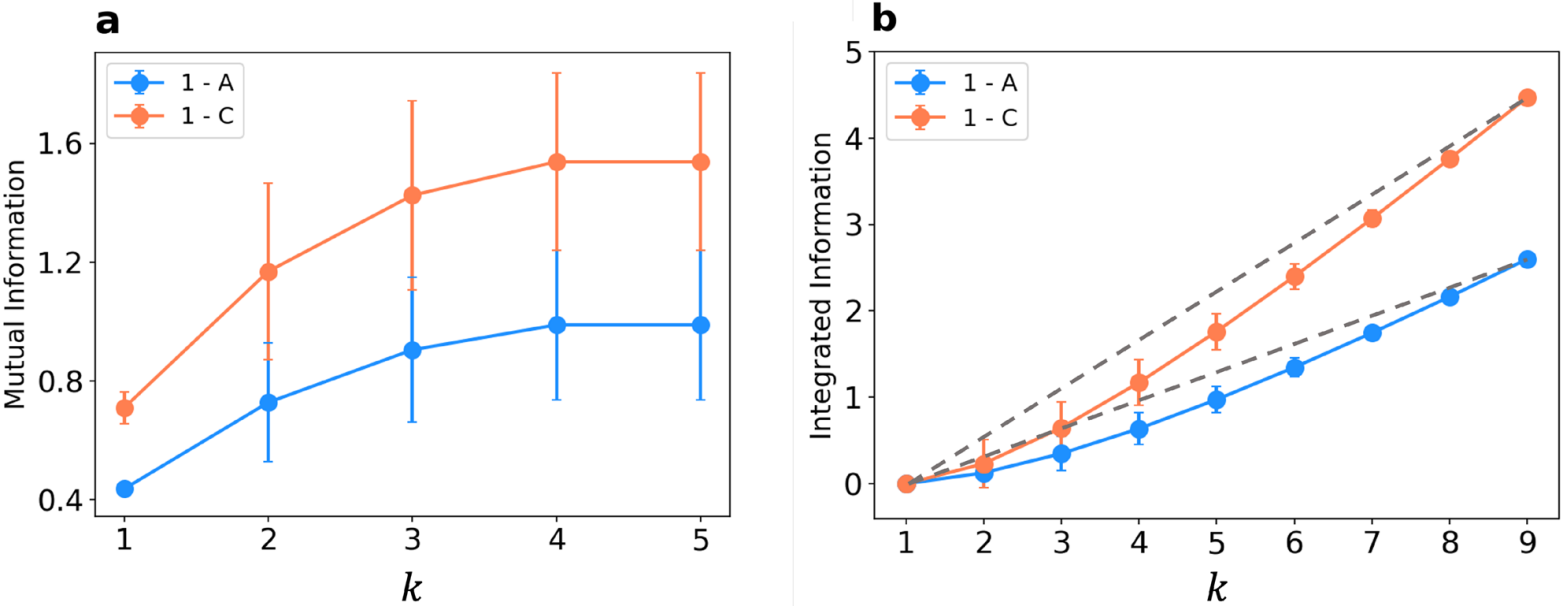
(**a**) experimental $\overline{\text {MI}}(k)$ for two different electrode couples to which $\Delta V=1$ V is applied (1-A: azure and 1-C: orange). Data are collected over 275 distinct configurations. (**b**) $\overline{\text {IN}}(k)$ for the same experimental data. Data are collected over 275 distinct configurations. In both cases, errorbars are given by the evaluation of these observables over all the different ways of choosing a given *k*. $\overline{\text {MI}}$ is only measured up to $k=5$, since $\text {MI}(X_j^k,X-X_j^k)=\text {MI}(X-X_j^k,X_j^k)$, while $\overline{\text {IN}}$ is measured for any $k \in [0,9]$. Grey dashed lines represent the $\overline{\text {IN}}$ growth in a fully correlated system.

Inspired by Refs.^21,39^, in order to understand how correlation between different regions of cluster-assembled films rises in response to the applied external voltage pulses, we experimentally measured MI and IN using a device shown in Fig. 7 and starting from the resistance time series of all the different electrode couples. In the following, we focus on data collected at $\Delta V=$1 V, after the conditioning stage (i.e. the application of a high voltage bias of 15 V). As an example, in Fig. 5a, $\overline{\text {MI}}$ is plotted as function of the number *k* of electrode couples, while applying the voltage bias either to the electrode couples 1-A (azure curves) or to 1-C (orange curves); Fig. 5b shows the equivalent result for $\overline{\text {IN}}$. $\overline{\text {MI}}$ and $\overline{\text {IN}}$ both show an increasing trend as a function of the subset size *k*. In particular, the Mutual Information monotonically increases, reflecting the correlations of a subset with its complementary subset. The application of $\Delta V$ to the electrode couple 1-C produces higher MI values: intuitively, in this configuration, the current flows throughout a larger region of the sample, probably enhancing reciprocal correlations among the sub-regions. Simultaneously, $\overline{\text {IN}}$ is larger when the voltage is applied to 1-C, mirroring the growing internal correlations among the elements of the chosen subset. In both cases, the growth of $\overline{\text {IN}}$ is sublinear, whereas a linear growth (represented as a grey dashed line in the picture) would indicate a fully correlated system. We observe a remarkable similarity with the trends usually observed in neuroscience works, where measurements are taken on animal brains^21,39^. Note that, in that field, the area comprised between the linear growth and the effective $\overline{\text {IN}}$ curve is a measure of the so called neural complexity.

**Figure 6:**
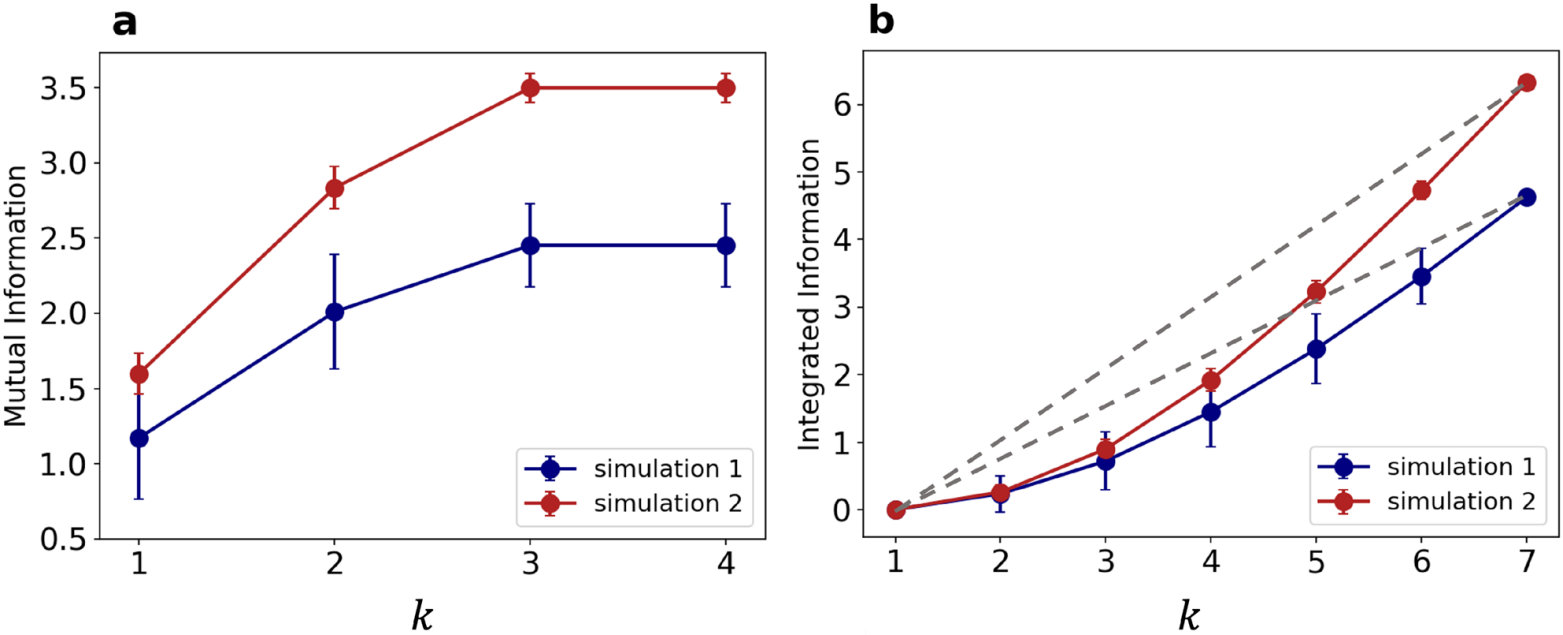
(**a**) $\overline{\text {MI}}(k)$ computed from two statistically independent simulations at $\Delta V=1$ V (blue and red), as a function of the number of subregions, *k*, considered. (**b**) $\overline{\text {IN}}(k)$ computed for the same simulations. In both cases, data are collected over 200 distinct MC configurations and errorbars are given by the evaluation of these observables over all the different ways of choosing a given *k*. Grey dashed lines represent the $\overline{\text {IN}}$ growth in a fully correlated system.

**Figure 7:**
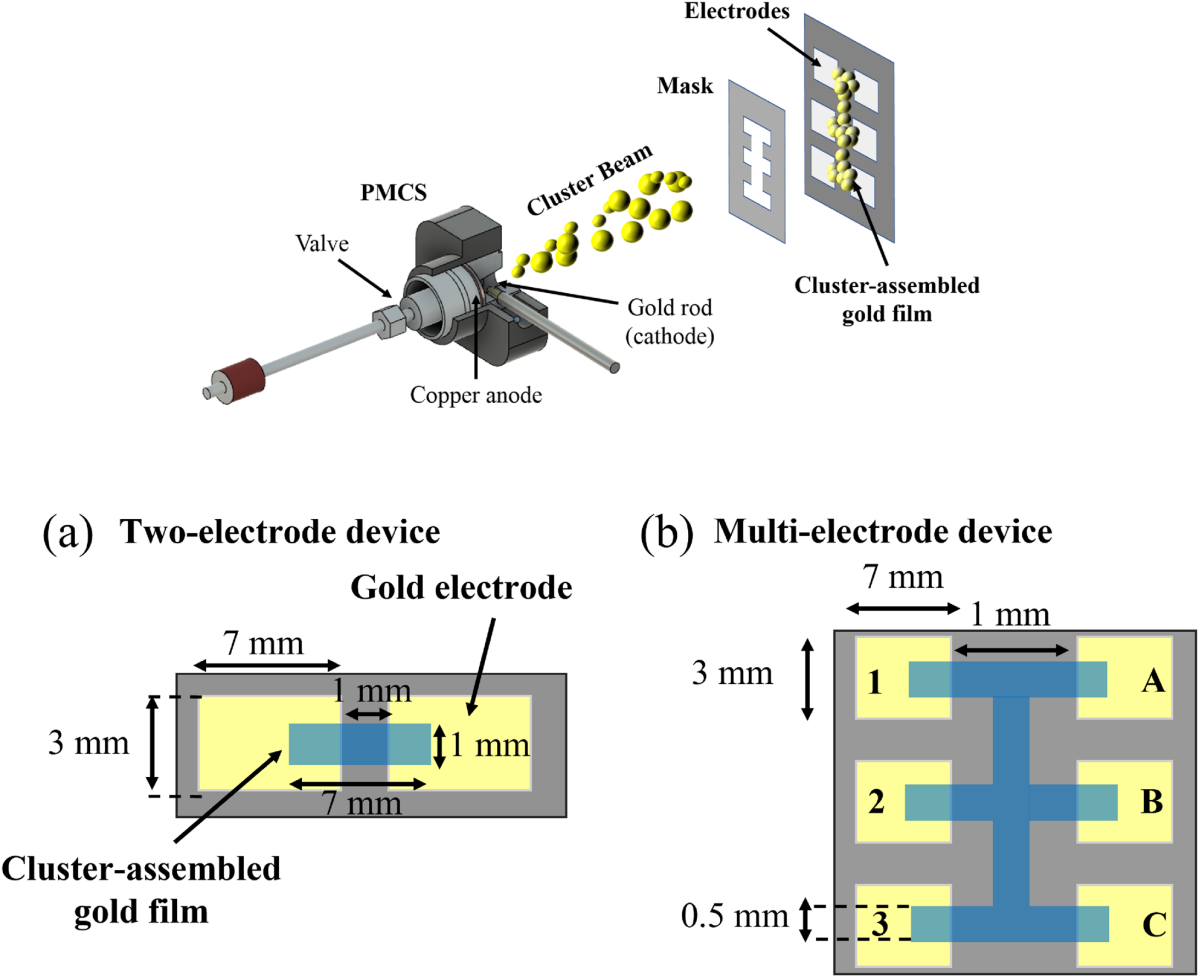
In the top panel the deposition process is depicted: the stencil masking allows the patterning of cluster assembled film via the interception of the cluster beam. In the bottom one two types of devices and their dimensions are depicted: (**a**) Two-electrode device, consisting of a couple of gold electrodes bridged by a cluster assembled Au film; (**b**) Multi-electrode device consisting of one vertical and three horizontal cluster-assembled film stripes linking the six electrodes.

In the complementary analysis of the simulations, the investigation of spatial correlations plays a crucial role as well. The topology of the nanostructured film just analyzed (see Fig. 7b) is more structured than the simulated network and it has more electrodes; therefore, the Information-theoretic tools based analysis in the SRN model necessarily requires a modification of the notion of $X_j^k$, which we choose to correspond to a well-defined sub-region of the network (see Fig. S4). In fact the network can be easily divided into *N* sub-regions (here we set $N=7$), each of them gathering the electrical properties of a number neighboring of links. The resistances of each sub-region (averaged over the links belonging to it) are periodically recorded in time, and their distribution is discretized with the same procedure employed for the experimental data (see Methods). In this way, it becomes possible to evaluate $\overline{\text {MI}}(k)$ and $\overline{\text {IN}}(k)$ even in the simulated system. In Fig. 6 we show $\overline{\text {MI}}(k)$ (panel a) and $\overline{\text {IN}}(k)$ (panel b) for two statistically independent simulations at $\Delta V=1$ V, after the application of a bias of 15 V. Statistical independence is guaranteed by the different initial configuration of conductances of the two SRNs used in simulations 1 and 2, which triggers a different electrical evolution for each of them. Remarkably, also $\overline{\text {MI}}(k)$ and $\overline{\text {IN}}(k)$ extracted from the SRN model simulations present some remarkable correlations among the various sub-regions of the whole simulated network. The growth trend of $\overline{\text {MI}}(k)$ and $\overline{\text {IN}}(k)$ as a function of the subsystem size *k* is exemplified in Fig. 6, and it is a general feature observed in all our simulations after the application of high voltage bias (see Supplementary Information for further discussion). Conversely, without the previous application of a high voltage, in experiments and as well in simulations we observe a substantial lack of dependence of $\overline{\text {MI}}(k)$ and $\overline{\text {IN}}(k)$ on *k*. Notably, the voltage which activates such behavior corresponds to the one which triggers the switching events, and, in the SRN model, promotes the reduction of the number of available shortest paths. Therefore, it turns out that a high $\Delta V$ bias is crucial to allow the system to visit neatly distinguished resistance states in the subsequent phase with a small applied tension. All these results suggest that the experimental and simulated systems have a complex and collective response to external stimuli, with an emerging electrical behavior determined by a subtle balance between the random dynamics and the reciprocal influence among different regions.

## Discussion

In this work, we have used an innovative approach named Stochastic Resistor Network model, designed at a coarse-grained level to reproduce some fundamental properties of cluster-assembled nanostructured gold films which can be exploited as neuromorphic devices^37^. By using a simple set of stochastic update rules for a set of electrical resistors arranged in a regular lattice, we were able to qualitatively mimic the complex electrical properties experimentally observed in cluster-assembled gold films. To achieve this, we found that the number of resistors in the network had to be considerably large. Our study therefore allowed us to obtain also an estimate of the minimum complexity needed to qualitatively recover the electrical transport properties in these nanostructured metallic films. The network required is particularly large; managing the computational workload to follow and characterize the stochastic evolution of the SRN model represents an unprecedented attempt in literature. We reported an extended analysis that compares the experimental and simulated resistive switching behavior: the trend of $R_{\mathrm{tot}}$, the ISI distribution, the PSD associated to $R_{\mathrm{tot}}$. Aiming to achieve a deeper understanding of the parallelism between our abstract model and the real system properties, we have gathered information about the evolution of current pathways within the network. The emergence of a peculiar behavior of the SRN model was interpreted in terms of a neat change in the relative abundance of links featuring each of the conductivity levels upon the application of different voltages. Leveraging Information Theory tools based on the information entropy related to the system conductive properties^21,39^, we analyzed both experimental and simulated systems with the goal to quantify the nontrivial spatial correlations detected among the different sub-regions. The dependence of the Mutual Information and of the Integrated Information from the size of the considered sub-region show some analogies with much more complex biological systems, for example the brain. Our results about the role and the evolution of spatial correlations strongly highlight the role non-linearity and non-locality in assemblies of simple electrical junctions. The nontrivial correlations emerging from the system dynamics the system dynamics suggests that SRN model simulations can be used to design neuromorphic devices based on complex networks of metallic nanoparticles^37,58^.

## Methods

### Cluster-assembled film fabrication

**Figure 8:**
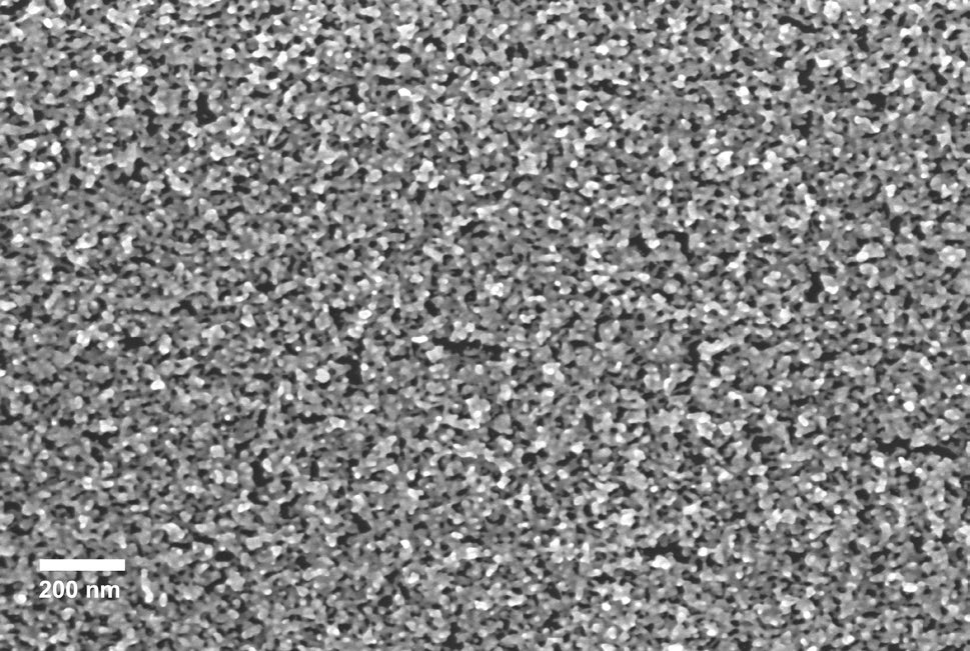
Scanning electron microscopy (SEM) micrograph of a cluster-assembled Au film deposited on Si substrate.

Cluster-assembled gold films were fabricated through Supersonic Cluster Beam Deposition (SCBD)^50^. This approach allows to control both the geometry of the deposited film and its electrical resistance, as described in detail in^26,37,50,51^. A Pulsed Microplasma Cluster Source (PMCS) produces neutral clusters in the gas phase by the ablation of a gold rod by a plasma ignited during the injection of a high-pressure pulse of Argon. The species resulting from the target ablation condense through collisions with the Argon gas forming Au clusters. The cluster-gas mixture is then expanded into a vacuum chamber, forming a supersonic seeded beam^26,50^. The cluster beam is focused by an aerodynamic lenses system^51^ and directed on an oxidized silicon substrate where gold electrodes are pre-deposited by thermal evaporation^37^. Two types of cluster-assembled films were fabricated: two-electrode and multi-electrodes systems (Fig. 7). Two-electrode films (Fig. 7a) consist of a couple of gold electrodes bridged by a cluster-assembled film, whereas multi-electrode systems consist of six gold electrodes connected by a cluster-assembled film shaped as a vertical stripe and three equally spaced horizontal stripes (Fig. 7b). The desired configuration is obtained by using a stencil mask^52^. The substrate holder is equipped with electrical contacts for the in situ characterization of the evolution of the electrical properties of the film during the deposition process. Cluster–assembled gold films beyond the percolation threshold (roughly 10 nm^57^) were fabricated in the range thickness 20–40 nm. Figure 8 reports a SEM micrograph of the typical morphology of a 20 nm thick cluster assembled gold film.

### Electrical characterization

The electrical measurements are carried out in the two probe configuration: a voltage bias of known value *V* is applied to one electrode, the second one being grounded, and the flowing current *I* is measured. The resistance *R* is computed as the ratio between the voltage and the current $R=V/I$. In the case of two-electrode device, a constant voltage is applied while *I* is sampled every 50 ms for a total time of around 1000 s (i.e., 20,000 points). In the case of multi-electrode devices, the measurement protocol is composed by a writing and a reading step. The former consists in an over-threshold pulse voltage train applied to one set of electrodes with a pulse amplitude in the range (−35V, 35 V) and width between 0.1 and 0.5 s. The latter comprises a train of sub-threshold voltage pulses, (1 V, width 0.05 s), which are applied in order to measure the resistance of all electrodes couples. This protocol allows to collect the evolution of resistance values of each electrode couple after the application of an over-threshold voltage pulse train. As result of the measurement a series of resistance data is associated to each electrode couple connected to the a region of the cluster assembled gold film.

### Resistance time series discretization in experiments and simulations

In both experiments and simulations, resistance time series $R_{\{t\}} (X_j^1)$ are collected. To build a probability distribution (from which entropy, MI and IN are computed), $R_{\{t\}} (X_j^1)$ is first of all normalized by its mean, $R'_{\{t\}} (X_j^1)=R_{\{t\}} (X_j^1)/\langle R_{\{t\}} (X_j^1) \rangle _t$. The normalized resistance value $R'_t (X_j^1)$ measured at time *t* is assigned to one of four discrete resistance levels, which are built considering the largest standard deviation $\delta _{max}$ among all those computed for the $\dfrac{N!}{k!(N-k)!}$ $R'_{\{t\}} (X_j^1)$ series, corresponding to the possible choices of a subset of size *k*, according to the following scheme:

6 $$\begin{gathered} R_{t}^{\prime } (X_{j}^{1} ) < \langle R_{{\{ t\} }} (X_{j}^{1} )\rangle _{t} - \delta _{{\max }} \hfill \\ \langle R_{{\{ t\} }} (X_{j}^{1} )\rangle _{t} - \delta _{{\max }} < R_{t}^{\prime } (X_{j}^{1} ) < \langle R_{{\{ t\} }} (X_{j}^{1} )\rangle _{t} \hfill \\ R_{{\{ t\} }} (X_{j}^{1} )\rangle _{t} < R_{t}^{\prime } (X_{j}^{1} ) < \langle R_{{\{ t\} }} (X_{j}^{1} )\rangle _{t} + \delta _{{\max }} \hfill \\ R_{t}^{\prime } (X_{j}^{1} ) > \langle R_{{\{ t\} }} (X_{j}^{1} )\rangle _{t} + \delta _{{\max }} \hfill \\ \end{gathered}$$

The histogram of the relative occurrences in each computed interval for each resistance series is thus built, constituting an approximation of the probability distribution of the simultaneous occurrence of the 4 computed states for the 9 electrode pairs (or for the 7 network sub-regions).

### Simulation methods

As shown in Fig. 2, our 3D network is modelled as a regular arrangement of resistors, represented as *links* *ij* joining pair of *nodes* (*i*, *j*). Links are organized in layers (*x*, *y* planes), stacked one on the top of each other. The largest network model we have characterized is made of $N_z = 3$ layers, each one containing $N_x \times N_y = 42 \times 27$ nodes. There are two special nodes, i.e. the *input* (source) and *output* (sink) ones; to these nodes it is applied a constant overall voltage $\Delta V$. The input/output nodes are connected to all the nodes in the first/last column of layer 1, respectively. Our network thus amounts to 3404 nodes and 8919 links, whose stochastic dynamics, simulated for tens of thousands of MC steps, necessarily requires massive use of parallel computing resources. Similar to^31,32^, we exploit spectral theory to compute the Laplacian matrix of the weighted undirected graph associated to the network^45–48^: the spectral decomposition of the Laplacian matrix $\mathbf {L}$ associated to the graph provides $\Delta V_{ij}$, $R_{ij}$ (and thus $I_{ij}$) at each edge and at each MC step, via the knowledge of the eigenvalues and eigenvectors of the Laplacian matrix^47^. Given a network configuration, the stochastic dynamics of the SRN model is obtained in the following way: first of all, we loop over all the network’s links, attempting to change their conductivity. For any link, its dissipated power $W_{ij}^{(d)} =\dfrac{\Delta V_{ij}^{2}}{R_{ij}}$ and the power that *ij* absorbs from its $N_{\mathrm{neigh }}$ neighbors $W_{ij}^{(a)} \propto \dfrac{1}{N_{\mathrm{neigh }}} \frac{\sum _{N_{\mathrm{neigh }}(k l)} \Delta V_{k l}^{2}}{R_{k l}}$ are computed, being *kl* the edges which are *ij*’s neighbors (here, chosen as those links which, together with *ij*, form elementary square plaquettes of the network). Therefore, an upgrade/downgrade of the conductance level of each link is proposed (and randomly accepted with a given probability); in particular, $W_{ij}^{(a)}$ ($W_{ij}^{(d)}$) is compared with a set of threshold power values, which fixes the smallest amount of absorbed (dissipated) power requested for an upgrade (downgrade) transition between two conductance levels. The exact thresholds and probabilities are summarized in Supplementary Information. Practically, we loop over all the network’s links and for each of them (see Supplementary for details):

1. if $\sigma _{ij} = \sigma _{\alpha }$, any trial modification necessarily attempts to increase $\sigma _{ij}$. If $\left| \Delta V_{i j}\right| >\Delta V^{\mathrm{th}}$ (being this a threshold voltage), try to upgrade $\sigma _{ij}$ to $\sigma _{\beta }$ with a probability $P_{\mathrm {nl}}$ to accept this nonlinear conductivity update. Otherwise, if $\left| \Delta V_{i j}\right| \le \Delta V^{\mathrm{th}}$, try to make $\sigma _{ij}$ become $\sigma _{\beta }/ \sigma _{\gamma }/ \sigma _{\delta }$ according to $W_{ij}^{(a)}$, with acceptance probability $P_{\mathrm{up}}.$
2. if $\sigma _{ij} = \sigma _{\beta }$ and if $\left| \Delta V_{ij}\right| < \Delta V^{\mathrm{th}}$, try to downgrade $\sigma _{ij}$ to $\sigma _{\alpha }$ with probability $P_{\mathrm {nl}}$. Else, if the condition for applying nonlinear gate is not satisfied, try to downgrade the link according to its $W_{ij}^{(d)}$, with probability $P_{\mathrm{down}}$. If, at this point, $\sigma _{ij}$ is still unchanged (due to the MC moves having been refused or to the low dissipated power), try to promote the link to become either $\sigma _{\gamma }$ or $\sigma _{\delta }$, with probability $P_{\mathrm{up}}$.
3. if $\sigma _{ij} = \sigma _{\gamma }$, first try to downgrade the link, down to either $\sigma _{\beta }$ or $\sigma _{\alpha }$, according to its $W_{ij}^{(d)}$. Last, in case the move is not accepted, try to upgrade it to $\sigma _{\delta }$, depending on $W_{ij}^{(a)}$ value.
4. if $\sigma _{ij} = \sigma _{\delta }$, the link can only be downgraded to become $\sigma _{\alpha }/ \sigma _{\beta }/ \sigma _{\gamma }$, according to its $W_{ij}^{(d)}$.

Importantly, we checked that the order of the trial updates is not relevant. With the just described algorithm, the conductance of each network edge changes at most once per step.

## Supplementary Information

Supplementary Information.

## Data Availability

The datasets generated during and/or analyzed during the current study are available from the corresponding author on reasonable request.
