# Supplementary material for: Dynamical stochastic simulation of complex electrical behavior in neuromorphic networks of metallic nanojunctions
Source: Sci Rep. 2022 Jul 18;12:12234. doi: 10.1038/s41598-022-15996-9 (PMC9294002; doi:10.1038/s41598-022-15996-9)
Supplement: Supplementary file 1 — Supplementary Information. [file 41598_2022_15996_MOESM1_ESM.pdf]

# Supplementary Information

# Dynamical stochastic simulation of complex electrical behavior in neuromorphic networks of metallic nanojunctions

F. Mambretti,[1,2] M. Mirigliano,[1] E. Tentori,[1] N. Pedrani,[1] G. Martini,[1] P. Milani*,[1] and D.E. Galli*[1]

[1]*Università degli Studi di Milano, CIMAINA and Dipartimento di Fisica, via Celoria 16, 20133 (Milano), Italy*

[2]*Università degli Studi di Padova, Dipartimento di Fisica e Astronomia, via Marzolo 8, 35131 (Padova), Italy*

## SUPPLEMENTARY NOTE: INPUT PARAMETERS AND MONTE CARLO SETTINGS

**Algorithm 1** SRNM: Monte Carlo update moves pseudo-code

**Require:** $W_{ij}^{(d)}$ and $W_{ij}^{(a)}$, $\forall ij$
**Require:** rnd is a uniformly distributed random number, $\in [0, 1)$
  **for** $ij \in$ network **do** ▷ loop over all netwrok's edges
    **if** $\sigma_{ij} = \sigma_\alpha$ **then**
      **if** $(|\Delta V_{ij}| > \Delta V^{\rm th})$ and (rnd $< P_{\rm nl}$) **then**
        $\sigma_{ij} \leftarrow \sigma_\beta$ ▷ try to apply nonlinear update move
      **else** ▷ try to apply thermal dissipation-based update move (a link with $\sigma_{ij} = \sigma_\alpha$ can only be upgraded)
        **if** $(W_{\rm up}^{th_1} + W_{\rm up}^{th_0} > W_{ij}^{(a)} \geq W_{\rm up}^{th_0})$ and (rnd $< P_{\rm up}$) **then**
          $\sigma_{ij} \leftarrow \sigma_\beta$
        **else if** $(W_{\rm up}^{th_2} + W_{\rm up}^{th_1} + W_{\rm up}^{th_0} > W_{ij}^{(a)} \geq W_{\rm up}^{th_1} + W_{\rm up}^{th_0})$ and (rnd $< P_{\rm up}$) **then**
          $\sigma_{ij} \leftarrow \sigma_\gamma$
        **else if** $(W_{ij}^{(a)} > W_{\rm up}^{th_2} + W_{\rm up}^{th_1} + W_{\rm up}^{th_0})$ and (rnd $< P_{\rm up}$) **then**
          $\sigma_\alpha \leftarrow \sigma_\delta$
    **else if** $\sigma_{ij} = \sigma_\beta$ **then** ▷ try to apply nonlinear update move
      **if** $(\Delta V^{\rm th} > |\Delta V_{ij}|)$ and (rnd $< P_{\rm nl}$) **then**
        $\sigma_{ij} \leftarrow \sigma_\alpha$
      **else** ▷ try to apply thermal dissipation-based update move
        **if** $(W_{ij}^{(d)} \geq W_{\rm up}^{th_0})$ and (rnd $< P_{\rm down}$) **then** $\sigma_{ij} \leftarrow \sigma_\alpha$
        **else if** $(W_{\rm up}^{th_2} + W_{\rm up}^{th_1} > W_{ij}^{(a)} \geq W_{\rm up}^{th_1})$ and (rnd $< P_{\rm up}$) **then**
          $\sigma_{ij} \leftarrow \sigma_\gamma$
        **else if** $(W_{ij}^{(a)} > W_{\rm up}^{th_2} + W_{\rm up}^{th_1})$ and (rnd $< P_{\rm up}$) **then**
          $\sigma_{ij} \leftarrow \sigma_\delta$
    **else if** $\sigma_{ij} = \sigma_\gamma$ **then**
      **if** $(W_{\rm up}^{th_1} + W_{\rm up}^{th_0} > W_{ij}^{(d)} \geq W_{\rm up}^{th_1})$ and (rnd $< P_{\rm down}$) **then** ▷ try to apply thermal dissipation-based update move
        $\sigma_\gamma \leftarrow \sigma_\beta$
      **else if** $(W_{ij}^{(d)} \geq W_{\rm up}^{th_1} + W_{\rm up}^{th_0})$ and (rnd $< P_{\rm down}$) **then**
        $\sigma_{ij} \leftarrow \sigma_\alpha$
      **else if** $(W_{ij}^{(a)} > W_{\rm up}^{th_2})$ and (rnd $< P_{\rm up}$) **then**
        $\sigma_\gamma \leftarrow \sigma_\delta$
    **else if** $\sigma_{ij} = \sigma_\delta$ **then**
      **if** $(W_{\rm down}^{th_2} + W_{\rm down}^{th_1} > W_{ij}^{(d)} \geq W_{\rm down}^{th_2})$ and (rnd $< P_{\rm down}$) **then**
        $\sigma_{ij} \leftarrow \sigma_\gamma$ ▷ try to apply thermal dissipation-based update move (a link with $\sigma_{ij} = \sigma_\delta$ can only be downgraded)
      **else if** $(W_{\rm down}^{th_2} + W_{\rm down}^{th_1} + W_{\rm down}^{th_0} > W_{ij}^{(d)} \geq W_{\rm down}^{th_1} + W_{\rm down}^{th_0})$ and (rnd $< P_{\rm down}$) **then**
        $\sigma_{ij} \leftarrow \sigma_\beta$
      **else if** $(W_{ij}^{(d)} \geq W_{\rm down}^{th_2} + W_{\rm down}^{th_1} + W_{\rm down}^{th_0})$ and (rnd $< P_{\rm down}$) **then**
        $\sigma_{ij} \leftarrow \sigma_\alpha$

Here follows a list of optimal parameters for the simulation input and for the MC moves.

- reasonable ranges of values for the (inital) abundance of each conductance are: $\sigma_\alpha$ : $1\% - 10\%, \sigma_\beta$ : $1\% - 10\%, \sigma_\gamma$ : $10\% - 30\%, \sigma_\delta$ : $50\% - 80\%$, for having a highly conductive network at the beginning of the simulation

- nonlinear move: $P_{\text{nl}} = 0.0003$, with a threshold potential of $0.06\,V$.

- thermal dissipation based moves: $P_{\text{up}} = P_{\text{down}} = 0.0015$ (probability to accept any move that changes $\sigma_{ij}$ upwards or downwards, regardless of the specific value of $\sigma_{ij}$):

  1. $\sigma_\alpha \leftrightarrow \sigma_\beta : W_{\text{up}}^{th_0} = 2 \times 10^{-6}$ J/s $= 2 \times W_{\text{down}}^{th_0}$
  2. $\sigma_\beta \leftrightarrow \sigma_\gamma : W_{\text{up}}^{th_1} = 10^{-6}$ J/s $= 2 \times W_{\text{down}}^{th_1}$
  3. $\sigma_\gamma \leftrightarrow \sigma_\delta : W_{\text{up}}^{th_2} = 2 \times 10^{-4}$ J/s $= 2 \times W_{\text{down}}^{th_2}$

  An example will immediately clarify the link update mechanism, according to its thermal dissipation: choose an edge $ij$, suppose that $\sigma_{ij} = \sigma_\delta = 4 \times 10^{-3}$ $1/\Omega$ and evaluate $W_{ij}^d$. If $W_{\text{down}}^{th_2} < W_{ij}^d \leq W_{\text{down}}^{th_1} + W_{\text{down}}^{th_2}$ the downgrade of $\sigma_{ij}$ value to $\sigma_\gamma = 2 \times 10^{-3}$ $1/\Omega$ is proposed (and accepted with probability $0.0015$). But if the dissipated power is large enough that $W_{\text{down}}^{th_2} + W_{\text{down}}^{th_1} + W_{\text{down}}^{th_0} > W_{ij}^d \geq W_{\text{down}}^{th_1} + W_{\text{down}}^{th_2}$, the link conductance will be decreased down to $\sigma_\beta = 10^{-3}$ $1/\Omega$ with probability $0.0015$, and so on. A completely identical mechanism holds for the absorbed power coming from each link neighbors. Obviously, links whose initial conductance is $\sigma_{ij} = \sigma_\alpha = 10^{-11}$ $1/\Omega$ can not be further downgraded.

Clearly, these particular choices of the threshold and probability values are not unique, but this set allows to approximately retrieve the essential experimental features in the simulated data, as largely discussed in the paper. Slight variations of these thresholds were found to generate analogous results.

## MINIMAL COMPLEXITY: NETWORK SMALL SIZE EFFECTS

Figure S1 represents $I(\Delta V)$ cycles performed with networks of different sizes, aiming to highlight the minimal complexity required for the qualitative reproduction of the experimental data. In these simulations, $\Delta V$ ranges from -8 V to 8 V, with 8000 MC steps for each applied voltage, and each network size has been investigated with 10 independent runs. If the network features a single layer, made by $N_x \times N_y = 32 \times 15$ nodes, the system presents a nonphysical behavior with wild current oscillations (see top panel).

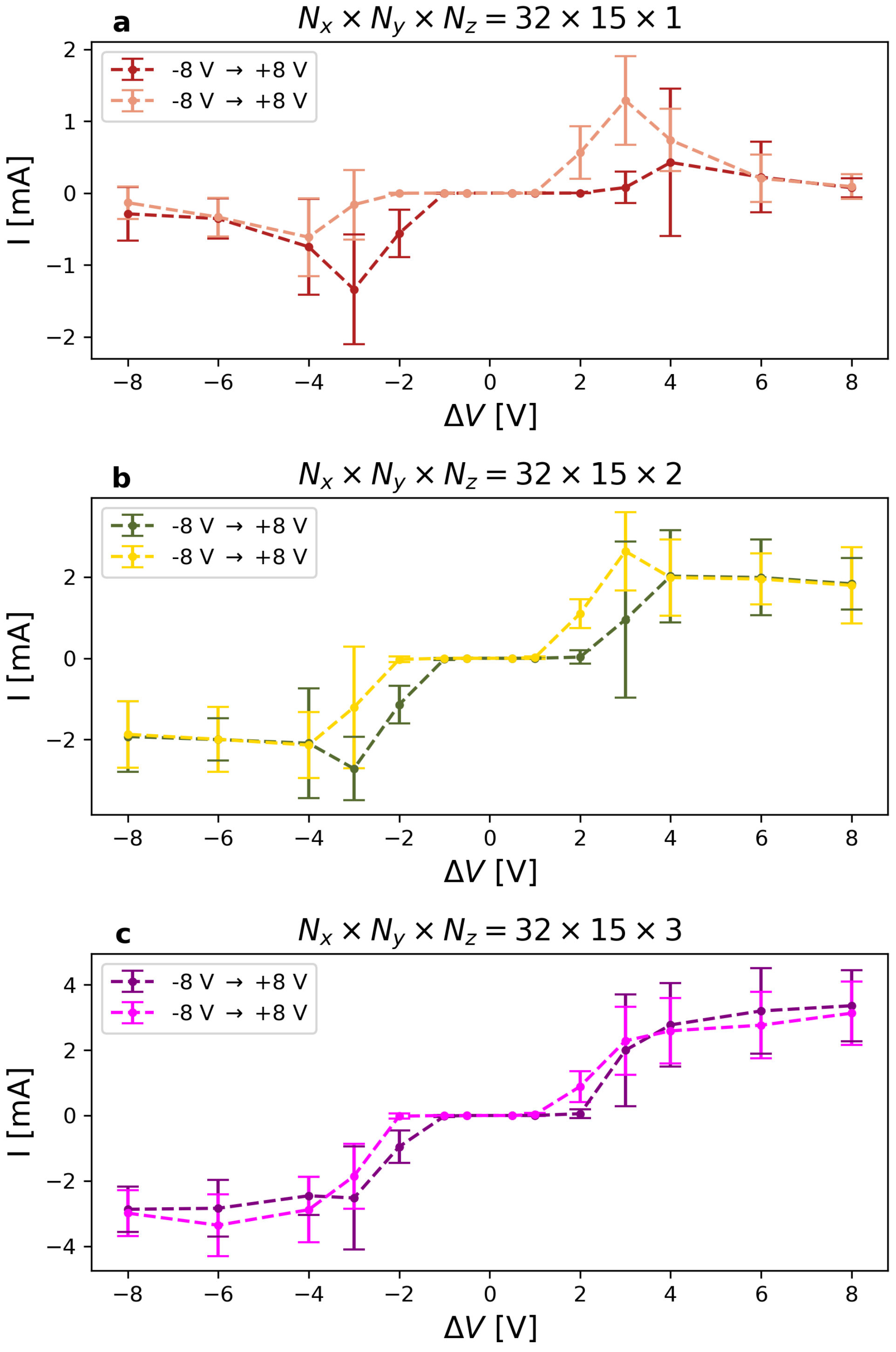


FIG. S1. $I(\Delta V)$ cycles, from -8 V to 8 V and back again to -8, simulated networks with different $N_x, N_y, N_z$ values. Top: $15 \times 32 \times 1$. Middle: $15 \times 32 \times 2$. Bottom: $15 \times 32 \times 3$. Errorbars are computed as the standard deviation of the mean over 10 independent runs.

The errorbars are almost anywhere extremely large, and a similar tendency is retrieved even

when a second layer is stacked on the top of the first one (panel **b**). This can be ascribed to the small amount of links in the network which, in turn, induce huge fluctuations in the availability of effective paths for the current to flow through the system. The addition of a third layer induces the onset of two distinct regimes, at low and high voltages, with a partial reduction of the errorbars (bottom panel). Fig. S1**c** begins to feature some similarities with the analogue experimental picture. We decided to use 3 layers and a much larger network ($N_x \times N_y = 42 \times 27$), with the aim to further reduce the errorbars onto the $I(\Delta V)$ curves and to provide more alternative paths for the input current to flow towards the output node. Nonetheless, the complexity of our system clearly remains much smaller than the experimental sample one, and this is - for instance - the origin of the current saturation at high voltages which is present in our SRN model, even in the largest simulated one.

## SUPPLEMENTARY NOTE: SHORTEST PATHS ANALYSIS

As mentioned in the main text, the structure and the topology of the network were studied via the investigation of the shortest path connecting the source and the sink of the system, thus exploring the possible paths in which the current could flow. This has been achieved thanks to the analysis we carried out with NetworkX tools [44]. We anticipate here that the application of a high voltage is found to dramatically reduce the number of alternative options to reach the output node.

What does it mean 'shortest'? Distances between pairs of nodes within a graph can be indeed measured, once that a metrics has been chosen. We consider here the $\mathbf{I}$ matrix ($N_n \times N_n$, i.e. $3404 \times 3404$) as a weighted graph for each MC step. Here we sketch the procedure, based on a Python custom code which integrates some NetworkX functions:

- load, for each time frame $t$ saved during the simulation, the corresponding matrices $\mathbf{I}|_t$ and $\mathbf{A}|_t$ into 2D `numpy` arrays.
- loop over $\mathbf{I}|_t$ elements: each entry $I_{ij}$ of $\mathbf{I}|_t$ corresponds to a weight $W_{ij} = \frac{1}{|I_{ij}|}$, if $A_{ij} \geq 10^{-3}\ 1/\Omega$, otherwise $W_{ij} = \infty$ (it is a sort of distance). The current values cover a wide range, typically between $10^{-11}A$ and $10^{-2}A$. The rationale behind the construction of this weight matrix $\mathbf{W}$ is that those links where the conductance is non–vanishing should be used by the current, while connecting node $0$ and node $N_\mathrm{n} - 1$. Conversely, highly (infinitely) resistive edges are essentially never crossed by the electrical current; assigning

them an infinite weight means excluding these edges (because the nodes they link are substantially disconnected) from the search of the SP connecting the source and sink nodes.

- once built the weight matrix $\mathbf{W}$, given two nodes $i$ and $j$, it is possible to calculate the shortest path between them, where the notion of distance is given by the weight matrix $\mathbf{W}$. NetworkX includes some methods for calculating the shortest path between a pair of nodes, in particular those by Dijkstra [10] and Bellman-Ford [11, 12].

Therefore, two quantities of interest can be defined:

- $L_{SP}$, i.e. the length of the shortest path, measured as an inverse current in $1/A$. We underline that, with this metrics, the shortest paths correspond to the most conductive source-drain pathways.

- $N_{SP}$, the number of equivalent shortest paths

In Figs. S2 and S3 we show, for a typical simulation, $L_{SP} \cdot \Delta V$ (which has the units of a resistance) and of $N_{SP}$ as a function of the MC step $t_{MC}$. On the one hand, it is evident that the length of the shortest path is strongly dependent on the applied voltage: Figure S2 shows the evolution of $L_{SP} \cdot \Delta V$ for a simulation with $\Delta V_{\text{tot}} = 1$ V (grey), before the application of the high voltage phase (green), which (at equilibrium, around 10000 MC steps) is very similar to the equilibrium $L_{SP}$ reached by the system at the same voltage *after* the conditioning step (blue).

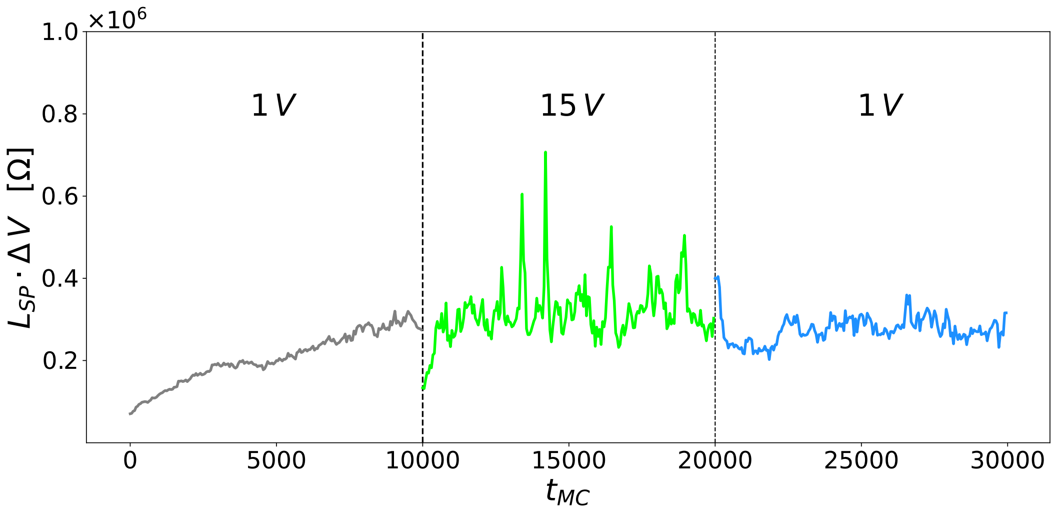


FIG. S2. $L_{SP} \cdot \Delta V(t_{MC})$, measured in $\Omega$, for a typical simulation. Grey: $\Delta V = 1$ V, green: $\Delta V = 15$ V, blue: $\Delta V = 1$ V after the writing phase.

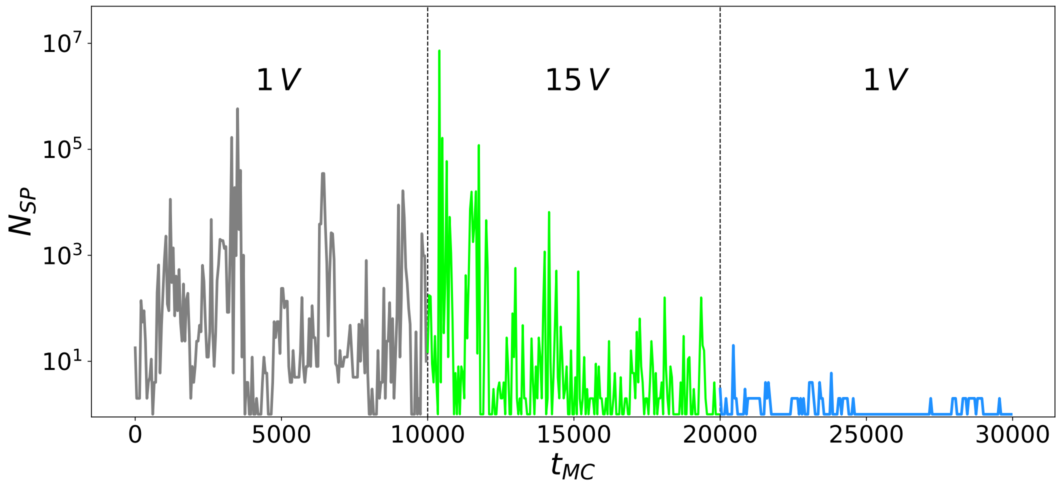


FIG. S3. $N_{SP}(t_{MC})$, for a typical simulation. Grey: $\Delta V = 1$ V, green: $\Delta V = 15$ V, blue: $\Delta V = 1$ V after the writing phase.

Conversely, the cardinality of the set of the shortest paths $N_{SP}$ is much different between the pre-conditioning and the post-conditioning phase. Note the $y-$log scale in Fig. S3: the grey curve signals the presence of a number of shortest paths comprised between 1 and $10^5$, approximately, while the blue curve always stays between 1 and 10. This means that the number of paths traversed by a huge amount of current is dramatically reduced by the application of $\Delta V = 15$ V. Figure 4 in the main text contains complementary information: the number of highly resistive links increases in the last part of the simulation, thus inducing a worsening of the percolative paths for current, reducing the options that easily connect the source and the sink.

## SUPPLEMENTARY NOTE: SPATIAL COARSE-GRAINING

In Fig. S4 it is shown the spatial coarse-graining method adopted for the network analysis via Information Theory tools. Black lines are the boundaries of a parallelepiped whose links are sent onto the coarse-grained region with index 7, as an example. An analogous procedure sends the other 6 subregions of the original system (left), onto the corresponding coarse-grained indexes (right). Note that also links connecting source/sink at the nods of the first layer participate into the spatial coarse-graining. Links of the original systems are colored according to their conductance, apart from the $\sigma_\alpha$ ones which are left white.

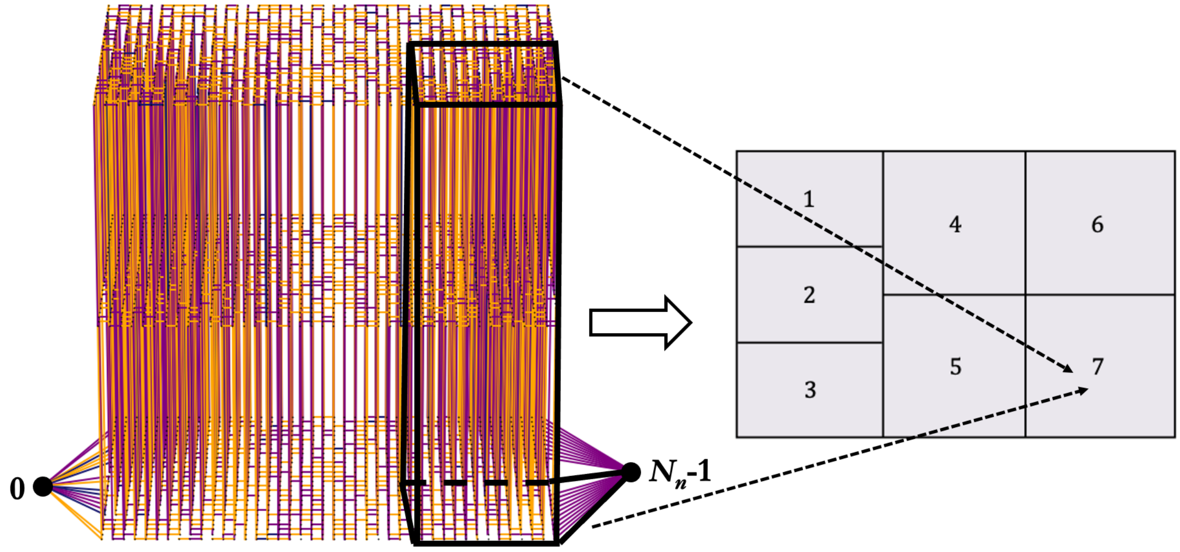


FIG. S4. Left: a typical snapshot of the resistor network, with link conductances colored with the same color scale used in Fig. 4. Realized with NetworkX [9]. Right: scheme of the spatially coarse-grained system obtained by dividing the network in 7 sub-regions. The sub-region of the original system which is mapped onto the coarse-grained group of index 7 is bounded by thick black lines.

## REFERENCES


[1] Kagan, M. On equivalent resistance of electrical circuits, *Am. J. of Phys.*, **83(1)**, 53–63 (2015)

[2] Rubido, N., Grebogi C., and Baptista, M. S. General analytical solutions for DC/AC circuit-network analysis. *Eur. Phys. J. - Special Topics* **226**, 1829–1844 (2017)

[3] Xiao, W. and Gutman, I. Resistance distance and Laplacian spectrum. *Theor. Chem. Acc.* **110(4)**, 284–289 (2003)

[4] Vishnoi, N. K. Lx = b Laplacian solvers and their algorithmic applications. *Found. Tr. Theor. Comp. Sci.* **8(1-2)**, 1–141 (2012)

[5] Moore, E.H. and Barnard, R. W. General Analysis. *Memoirs of the American Philosophical Society*, **I**, 1, 197–209 (1935)

[6] Penrose, R., A generalized inverse for matrices. *Mathematical Proceedings of the Cambridge Philosophical Society*, **51**, 3, 406-413 (1955)

[7] Sanderson, C. and Curtin, R., Armadillo: a template-based C++ library for linear algebra. *JOSS* **1(2)**, 26 (2016)

[8] Sanderson, C. and Curtin, R., A User-Friendly Hybrid Sparse Matrix Class in C++. *Mathematical Software – ICMS 2018* **10931** (2018)

[9] Hagberg, A. A., Schult, D. A. and Swart P. J., Exploring network structure, dynamics, and function using NetworkX. *Proc. 7th Python Sci. Conf. (SciPy2008)*, 11–15, (2008)

[10] Dijkstra, E., A note on two problems in connexion with graphs. *Numerische Matematik*, **1**, 1, 269-271 (1959)

[11] Bellman, R., On a routing problem. *Quarterly of Applied Mathematics* **16**, 1, 87-90 (1958)

[12] Ford, L., Network Flow Theory, 1-12 (1956)
